# Enhanced performance of an innovative dengue IgG/IgM rapid diagnostic test using an anti-dengue EDI monoclonal antibody and dengue virus antigen

**DOI:** 10.1038/srep18077

**Published:** 2015-12-11

**Authors:** Jihoo Lee, Young-Eun Kim, Hak-Yong Kim, Mangalam Sinniah, Chom-Kyu Chong, Hyun-Ok Song

**Affiliations:** 1 Department of Biochemistry, College of Natural Sciences, Chungbuk National University, Cheongju, Chungbuk, Republic of Korea; 2 GenBody Inc., Biotech Business IC, Dankook University, Cheonan, Chungnam, Republic of Korea; 3 Hospital Kuala Lumpur, Jalan Pahang, Malaysia; 4 Department of Infection Biology, Wonkwang University School of Medicine, Iksan, Jeonbuk, Republic of Korea

## Abstract

High levels of anti-dengue IgM or IgG can be detected using numerous rapid diagnostic tests (RDTs). However, the sensitivity and specificity of these tests are reduced by changes in envelope glycoprotein antigenicity that inevitably occur in limited expression systems. A novel RDT was designed to enhance diagnostic sensitivity. Dengue viruses cultured in animal cells were used as antigens to retain the native viral coat protein. Monoclonal antibodies (mAbs) were then developed, for the first time, against domain I of envelope glycoprotein (EDI). The anti-dengue EDI mAb was employed as a capturer, and EDII and EDIII, which are mainly involved in the induction of neutralizing antibodies in patients, were fully available to bind to anti-dengue IgM or IgG in patients. A one-way automatic blood separation device prevented reverse migration of plasma and maximize the capture of anti-dengue antibodies at the test lines. A clinical evaluation in the field proved that the novel RDT (sensitivities of 96.5% and 96.7% for anti-dengue IgM and IgG) is more effective in detecting anti-dengue antibodies than two major commercial tests (sensitivities of 54.8% and 82% for SD BIOLINE; 50.4% and 75.3% for PanBio). The innovative format of RDT can be applied to other infectious viral diseases.

Dengue is a mosquito-borne viral infection that causes a flu-like illness and occasionally develops into severe diseases, such as dengue hemorrhagic fever and dengue shock syndrome^1^. Infection with one of the four serotypes of dengue virus (DENV1–4) is typically asymptomatic or mildly symptomatic^1^,^2^,^3^, but a secondary infection with a different serotype of DENV can cause severe disease^1^,^4^,^5^. The incidence of dengue is continuously increasing around the world, particularly in the tropics and subtropics, which are favorable for the growth of vector mosquitos, e.g., *Aedes aegypti* and *A. albopictus*^6^. Recent reports estimate that there are 390 million dengue infections per year in 128 countries, of which 96 million show clinical manifestations with any disease severity^7^,^8^. Although dengue is a major global public health problem, there is no specific treatment for dengue fever and no licensed vaccine to protect against dengue. Rapid and accurate diagnosis, therefore, is important to control dengue-related diseases.

Serological diagnosis of dengue relies on the detection of high levels of anti-dengue IgM or anti-dengue IgG, which recognize and bind to DENV antigen. Envelope glycoprotein (E), a structural protein of DENV^9^, is the most important antigenic target of these neutralizing antibodies because it is exposed on the viral surface and mediates both host cell receptor binding^10^ and fusion^11^. The E protein consists of three domains, which are distinct in structure and function, i.e., envelope domain I (EDI), EDII, and EDIII^12^. EDIII, in particular, contains serotype-specific and conformation-specific neutralization epitopes^10^,^13^,^14^,^15^,^16^,^17^,^18^. The antibody against EDIII indeed inhibits the receptor binding of viruses to host cells^10^,^17^,^19^,^20^ and thereby is of interest with respect to the development of a subunit vaccine as well as an antiviral antibody^21^,^22^,^23^,^24^,^25^,^26^. EDII also contains serotype-conserved epitopes that induce neutralizing antibodies related to structural changes that affect virus–cell membrane fusion^10^,^13^,^21^,^27^,^28^. Antibodies against epitopes in EDI are not neutralizing^13^,^17^,^20^. EDI is less immunogenic than EDII and EDIII, and it has a major role in the dimeric structural organization of the E protein^29^.

The sensitivity and specificity of serological assays for detecting anti-dengue IgM and anti-dengue IgG are strongly influenced by antigen quality. Currently, viral antigens are prepared by 1) generating a recombinant envelope protein, typically EDIII or a EDII/EDIII fusion protein or 2) expressing virus-like particles (VLPs) in a baculovirus vector-insect cell system. However, the recombinant DENV envelope protein expressed in a general bacterial system tends to lose its antigenic properties, such as its native structure and glycosylation pattern. This can lead to false-negative results and a decreased assay sensitivity. Reactivity is increased when a VLP is used as an antigen, but it is difficult to produce VLPs in large quantities. In addition, the protein glycosylation of baculovirus-infected insect cells is not equivalent to that of mammalian cells^30^,^31^,^32^,^33^. Thus, a decrease in diagnostic sensitivity is inevitable.

In this study, novel strategies to improve the diagnostic sensitivity and specificity were developed. Entire DENV particles were cultured in animal cells, purified, and used as antigens. This method does not involve modification of viral structure, including glycosylation patterns; accordingly, it is possible to obtain a highly immunogenic antigen. To maximize the binding of anti-dengue IgM or anti-dengue IgG to the DENV antigen, mAbs against EDI were developed and used to hold the DENV antigen. As EDII and EDIII of DENV particles are freely available, it is possible to detect anti-dengue IgM or anti-dengue IgG with high sensitivity. A rapid immunochromatographic test was developed using these strategies and was clinically evaluated.

## Results

### Generation of mAbs against the peptide antigen of DENV EDI

mAbs were generated against the peptide antigen of DENV EDI. The peptide sequences were selected as predictions suggested that the sequences have few or no glycosylation sites and they do not contribute to the conformational arrange of the protein. The selected sequences were well conserved among 4 serotypes of dengue virus but not in the other flaviviruses such as Japanese encephalitis virus, Tick-borne encephalitis virus, and Yellow fever virus as well as in the alphavirus such as Chikungunya virus. Hybridoma cell lines stably producing independent mAbs were established, and were screened using an enzyme-linked immunosorbent assay (ELISA). Six cell lines showing high reactivity with the peptide antigen were selected: M5G6, M1G10, M1E4, M3F2, M3G8, and M4D12. Among them, 4 hybridoma cell lines exhibited high reactivity with DENV cultured in animal cells: M5G6, M1G10, M1E4, and M3F2. The dissociation constant (K_d_) was determined by measuring the affinity of each mAb to both the peptide antigen and animal-cell cultured virus, and generating a Klotz plot (Table 1). Isotypes of mAbs were identified as IgG_1_ (M5G6, M1G10, and M3F2), IgG_2a_ (M3G8 and M4D12), or IgG_2b_ (M1E4).

### Design of the dengue IgG/IgM rapid test with a novel format

**Figure 1:**
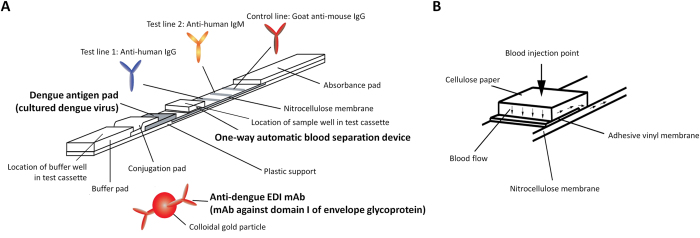
The novel dengue IgG/IgM rapid test strip. (**A**) The construction of the strip with a novel design; (**B**) one-way automatic blood separation device.

**Figure 2:**
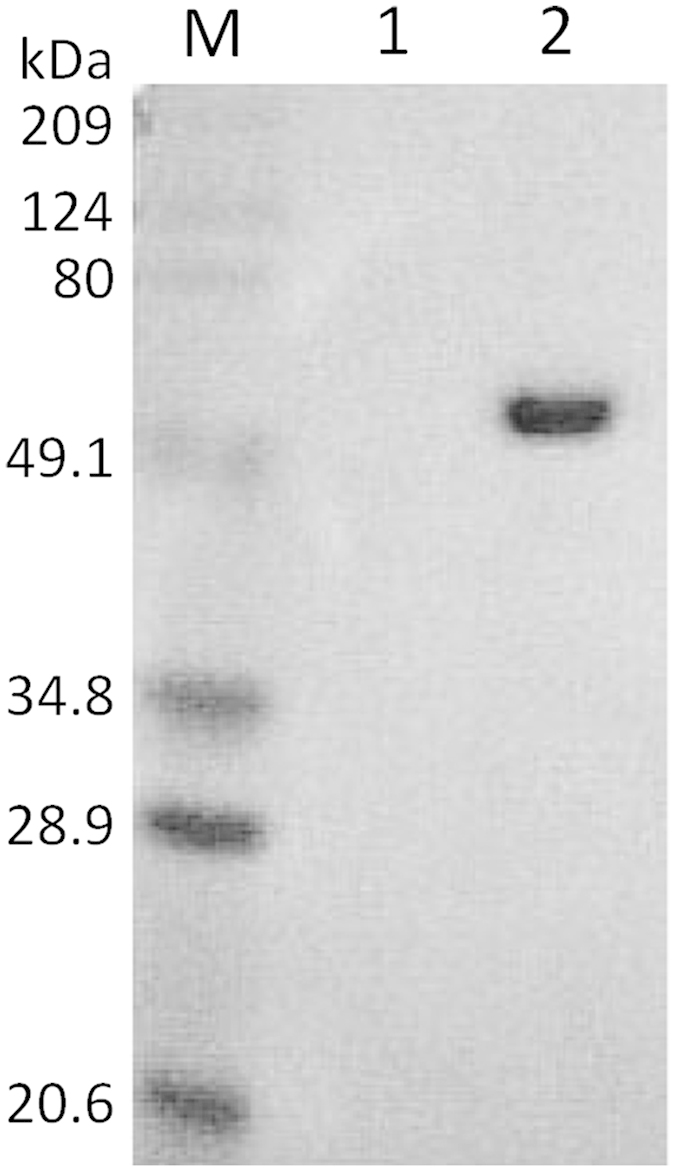
Reactivity of the monoclonal antibody M5G6 to animal cell-cultured dengue virus particles was confirmed by a western blot analysis. M, size marker; 1, Hepatitis B virus; 2, Dengue virus cultured in and purified from animal cells.

**Figure 3:**
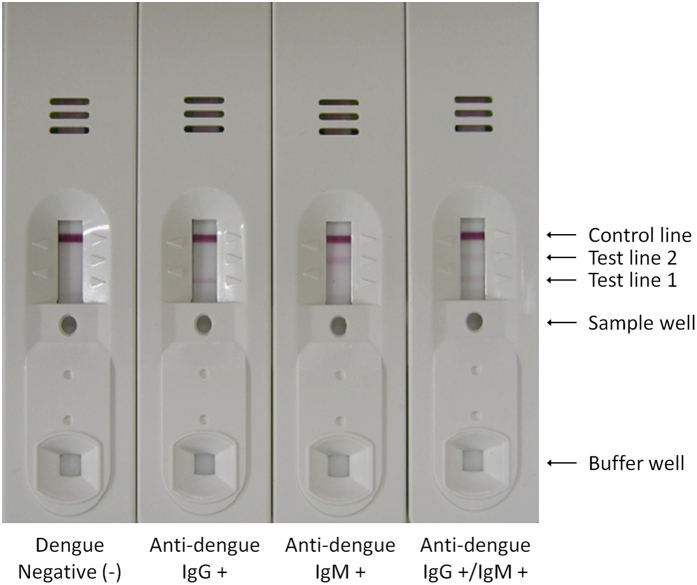
The novel dengue IgG/IgM rapid test strip in a plastic cassette. The diagnostic results are shown with dengue-negative, anti-dengue IgG-positive, anti-dengue IgM-positive, and anti-dengue IgG- and IgM-double positive samples.

The rapid test used a novel format in order to simultaneously detect both anti-dengue IgG and anti-dengue IgM in infected blood with high sensitivity and specificity. Basically, a test configuration is similar to what is used in other test kits. As a means of improving the diagnostic sensitivity and accuracy, the rapid test was equipped three unique devices, the DENV antigen pad, the conjugator pad containing anti-dengue EDI mAb (M5G6), and the sample pad, which enabled one-way automatic blood separation (Fig. 1). The DENV antigen pad was prepared by treating DENV purified from animal cell cultures. This method can deliver a highly immunogenic antigen as it does not involve modification of viral structure, including glycosylation patterns, which inevitably occurs in the preparation of recombinant antigens. Antigen pad was placed in close proximity to the conjugator pad, which contained anti-dengue EDI mAb (M5G6) conjugated with colloidal gold particles (Fig. 1A). Thus, after buffer solution is applied to the buffer well, upon migration, the mAb-gold conjugate can capture DENV particles owing to its high and specific reactivity to virus particles (Fig. 2). These captured DENV antigens can be recognized by anti-dengue IgG or anti-dengue IgM in blood samples. Since DENV is captured by the mAb-gold conjugate via EDI, EDII and EDIII of DENV particles are freely available for antibody binding. Therefore, it is possible to obtain maximum binding of anti-dengue IgG or anti-dengue IgM to DENV antigens and a highly sensitive test. The specific sample pad in this novel rapid test enables the automatic separation of whole blood components owing to the asymmetric structure of the Vivid membrane (Fig. 1B). Whole blood is applied to the sample well of the test kit, and only plasma separates and is able to migrate along the nitrocellulose membrane of the strip. Unlike any other rapid tests, blood flow in this novel rapid test is guided only toward test lines via an adhesive vinyl membrane that is intentionally placed partially toward the conjugator pad (Fig. 1B). This prevents reverse migration of plasma in the strip and thereby, anti-dengue antibodies in plasma can entirely react with anti-human IgG or anti-human IgM antibodies immobilized at the test lines. By positioning this one-way automatic blood separation device closer to the test lines of the strip than to the DENV antigen pad, anti-dengue IgG or anti-dengue IgM in blood samples are first recognized and captured by anti-human IgG or anti-human IgM antibodies at the test lines. After the addition of buffer solution, the anti-dengue EDI mAb-gold conjugate is released from the conjugator pad, and it continuously captures DENV antigens while migrating through the DENV antigen pad. DENV antigen-anti-dengue EDI mAb-gold conjugate complex migrates along the nitrocellulose membrane because migration toward the sample pad is inhibited by an adhesive vinyl membrane. The complex then meets anti-dengue IgG or anti-dengue IgM captured at the test lines and the final complex, i.e., the anti-dengue IgG or anti-dengue IgM-DENV antigen-anti-dengue EDI mAb-gold conjugate, is formed at the test line. The formation of the final complex is visualized at the test line by the color of colloidal gold to determine positivity (Fig. 3). Unbound anti-dengue EDI mAb-gold conjugate migrates to the control line, and it is captured by anti-mouse IgG. Thus, the color should appear at the control line in all valid tests (Fig. 3). Similar to other kits, anti-human IgM is placed at test line 2, which is relatively far from the sample well and thereby requires more time to reach, i.e., it has more time to react. Anti-dengue IgM is produced at the early stage of DENV infection; thus, more sensitive and accurate detection of anti-dengue IgM is required.

### Validation of the novel RDT by a comparative evaluation

**Figure 4:**
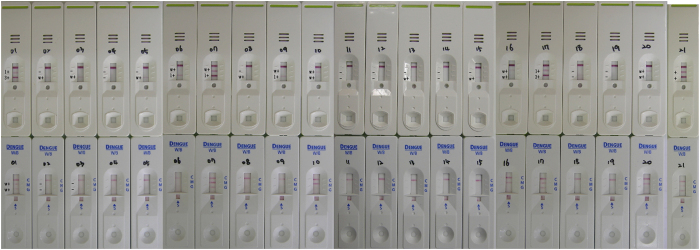
Validation of the novel dengue IgG/IgM rapid diagnostic test (RDT) by a comparative evaluation. An anti-dengue mixed titer performance panel was used as an evaluation panel. Upper panel, novel dengue IgG/IgM RDT; lower panel, SD BIOLINE dengue IgG/IgM RDT. Serial numbers of clinical specimens are shown on each device.

**Figure 5:**
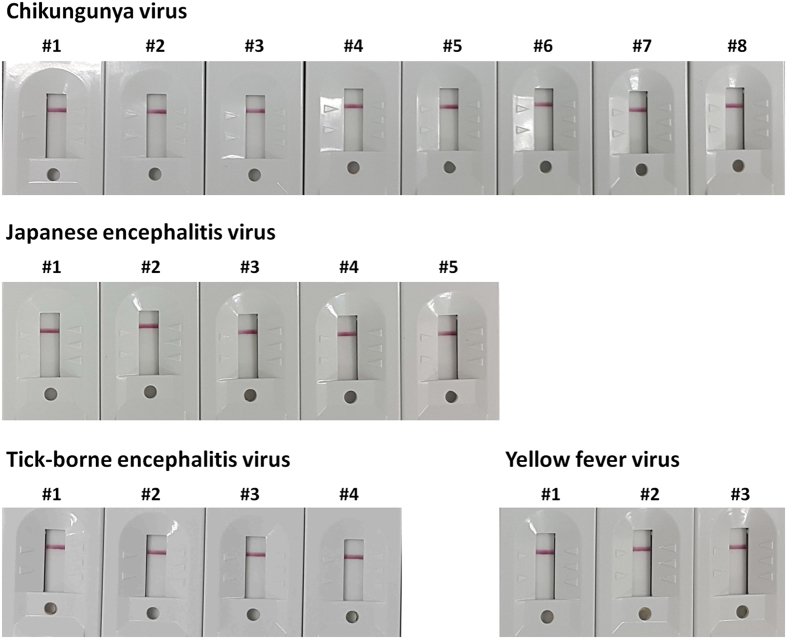
No cross-reactivity was shown with an alphavirus and other flaviviruses. Chikungunya virus-, Japanese encephalitis virus-, Tick-borne encephalitis virus-, and Yellow fever virus-infected clinical specimens were tested by the novel dengue IgG/IgM RDT. All specimens exhibited color signals only at the control line.

The diagnostic efficiency of the novel dengue IgG/IgM rapid test was validated using an evaluation panel tested by various immunological assays that are available on the market in a special program sponsored by the World Health Organization^34^. The evaluation panel was tested by 6 ELISAs, 3 rapid tests, and 1 immunofluorescence assay. DENV serotypes in the evaluation panel were determined by an immunofluorescence assay and real-time RT-PCR^35^. The diagnostic results of the novel dengue IgG/IgM rapid test were compared to those of SD BIOLINE Dengue IgG/IgM (Alere Inc., Waltham, MA, USA), which is widely used worldwide. Based on the reactivities for anti-dengue IgG and IgM antibodies obtained from SD Dengue IgG or IgM capture ELISA, the sensitivity and specificity of both RDTs were calculated. The commercial kit showed 9 false-negatives for anti-dengue IgG-positive samples (nos. 2, 3, 5, 6, 8, 9, 10, 11, and 13) and 3 false-negatives for anti-dengue IgM-positive samples (nos. 6, 8, and 9), indicating sensitivities of 40% (6/15) and 70% (7/10), respectively (Table 2 and Fig. 4). The observed specificities were 100% for negative samples. The novel dengue IgG/IgM rapid test that was developed in this study showed considerably higher sensitivities than those of the commercial test, i.e., 93.3% (14/15) and 100% (10/10) against anti-dengue IgG-positive and anti-dengue IgM-positive samples, respectively. It generated only 1 false-negative (no. 11) and 1 false-positive (no. 15) result from anti-dengue IgG-positive samples, and 1 false-negative from anti-dengue IgM-positive samples (no. 8) (Table 2 and Fig. 4). The specificities were 83.3% (5/6) and 100%, respectively. The novel dengue IgG/IgM rapid test was able to detect all DENV serotypes; several specimens diagnosed as positive by the kit were single-infected with DENV-4 (nos. 1, 2, 3, 5, and 6), DENV-3 (nos. 16 and 17), and DENV-1 (nos. 13 and 20)^35^. No cross reactivity was shown with other flaviviruses such as Japanese encephalitis virus, Tick-borne encephalitis virus, and Yellow fever virus as well as with an alphavirus such as Chikungunya virus (Fig. 5).

### Clinical evaluation of the novel RDT

The novel dengue IgG/IgM rapid test was further evaluated using clinical specimens collected from Kuala Lumpur General Hospital, Malaysia. Sensitivities of 100% (320/320) and 99.5% (189/190) were observed for anti-dengue IgG-positive and anti-dengue IgM-positive blood samples (Table 3). One false-positive (positive in both anti-dengue IgG and IgM) result was obtained using the novel kit, leading to a specificity of 99.3% (149/150) (Table 3).

A field evaluation was then performed using the novel RDT in Malaysia, an endemic area for dengue. Two common commercial RDTs were used for a comparative evaluation. A total of 320 blood specimens collected between September 2011 and January 2012 were tested: 113 anti-dengue IgM-positive, 150 anti-dengue IgG-positive, and 57 dengue-negative samples (Table 4). The novel RDT developed in this study exhibited a sensitivity of 96.5% (109/113) for anti-dengue IgM-positive specimens. The commercial tests showed sensitivities of 54.9% (62/113 for SD BIOLINE) and 50.4% (57/113 for PanBio), respectively. Interestingly, the novel RDT was able to detect secondary DENV infections more effectively than commercial products. Thirty-six of 113 anti-dengue IgM-positive samples were also positive for anti-dengue IgG using the novel RDT, leading to a sensitivity of 31.9% for the detection of secondary dengue infection. The SD BIOLINE and PanBio products detected 23 and 27 specimens with secondary dengue infections, resulting in sensitivities of 20.4% and 23.9%, respectively. For anti-dengue IgG-positive specimens, the novel test had a sensitivity of 96.7% (145/150), but those of the commercial tests were 82.0% (123/150 for SD BIOLINE) and 75.3% (113/150 for PanBio), respectively. The specificity of the RDTs were all 100%. All 57 dengue-negative specimens were negatively diagnosed using the 3 RDTs. The cross-reactivity of the RDTs was tested using 15 blood samples obtained from non-dengue viral infections, including cytomegalovirus, measles, rubella, Epstein–Barr virus, and human parvovirus B19 acute infection. No cross-reaction was observed for the 3 RDTs used in the evaluation.

## Discussion

A novel dengue IgG/IgM rapid test was developed in this study. It displayed excellent diagnostic capability. The sensitivity and specificity of the test were considerably higher than those of rapid tests that are currently available on the market. Previously, anti-dengue IgM rapid tests have been evaluated by the World Health Organization^34^. These evaluations have been conducted using anti-dengue IgM-positive serum specimens collected at 7 locations worldwide (Thailand, Cambodia, Malaysia, Vietnam, Puerto Rico, Argentina, and Cuba). Test kits include the dengue duo cassette (PanBio Diagnostics, Alere Inc.), Dengucheck WB (Zephyr Biomedicals, Dona Paula, India), and SD dengue IgG/IgM (Standard Diagnostics, Alere Inc.). The mean sensitivities of these commercial kits were 77.8%, 20.5%, and 60.9%, respectively. The mean specificities were 90.6%, 86.7%, and 90%, respectively. The novel kit developed in this study displayed superior sensitivity (99.5–100%) and specificity (99.3–100%) against various international dengue-positive specimens (Colombia, Ecuador, Honduras, and Malaysia) (Tables 2 and 3). However, a direct comparison among rapid test kits is required using identical clinical specimens.

The novel kit was characterized by substantial improvements in sensitivity and specificity owing to its strategic organization, complementing both properties. It uses DENV itself, rather than recombinant proteins, as an antigen, and this maintains the native antigenicity of the virus antigen. There are no artificial changes to virus antigens; accordingly, the structural properties of the antigen, such as its glycosylation pattern, are preserved. In addition, the anti-dengue EDI mAb does not interfere with the binding of anti-dengue IgG or anti-dengue IgM to the EDII and EDIII domains, which are the major binding sites of immunoglobulins produced against DENV in patients. These strategies indeed maximized the sensitivity and accuracy of the rapid test. Besides, a one-way automatic blood separation device prevents the reverse migration of plasma in the strip and lead to the maximum binding of anti-dengue antibodies in plasma to anti-human IgG or anti-human IgM antibodies at the test lines.

A field evaluation proved that the novel RDT is far more sensitive and accurate for the detection of both anti-dengue IgG and anti-dengue IgM than commercial tests currently used in the field (Table 4). In particular, the ability of the novel kit to detect anti-dengue IgM was superior. The sensitivity was almost twice those of the commercial kits. Thus, the novel RDT developed in this study is very useful and is suitable for the diagnosis of acute dengue infection via the detection of dengue-specific IgM antibodies. In a large laboratory that conducts automated ELISA to detect anti-dengue IgM as a first-line test, the novel RDT developed in this study might be helpful as a rapid secondary test to differentiate primary from secondary dengue infection. The use of this novel RDT can accelerate dengue diagnosis in such large and busy laboratories. Additionally, it may be useful as a screening test in a small laboratory setting that lacks modern facilities for automated dengue testing. The RDT enables such laboratories to detect acute dengue and to differentiate primary from secondary DENV infection in 15 min. At present, there are no commercial dengue-specific IgG ELISA tests for the detection of low/background IgG antibodies; the novel RDT can be used for this function owing to its highest sensitivity for anti-dengue IgG among the 3 RDTs evaluated in this study. The PanBio ELISA for anti-dengue IgG is designed to capture only high levels of IgG i.e., an HAU titer of 1:2,560, the titer used to distinguish secondary DENV infection from primary or past DENV infection^36^,^37^. Therefore, it cannot discriminate all cases of dengue. In this context, the novel RDT developed in this study has a great advantage as an *in vitro* diagnostic tool for discrimination. In addition, it can be used as a screening test to estimate the prevalence of dengue-specific IgG in population-based studies or in epidemiologic studies in the field.

In summary, a highly sensitive and accurate rapid test was developed for dengue detection and its use was clinically validated in the field. The rapid test was established using a novel design that applies DENV particles directly as antigens to maximize diagnostic sensitivity and to minimize false-negative results. This was achieved using mAbs that were specific to EDI of DENV, which have never previously been developed. In addition, the rapid test included a specific device (a one-way automatic blood separation device) to induce a maximum capture of anti-dengue antibodies at the test lines by preventing reverse migration of plasma. Clever positioning of all components (i.e., an antigen pad, a sample pad, a conjugation pad, and immobilized antibodies) of the test kit also helped improve the diagnostic sensitivity. Based on a clinical field evaluation, this novel rapid test was highly effective in detecting viral antibodies, and thus the test format can be applied to other infectious viral diseases.

## Methods

### Preparation of peptide antigen

A peptide containing the domain I sequence of the dengue virus serotype 2 envelope protein was synthesized (Peptron Inc., Daejeon, Korea); N′-TGHLKCRLRMDKLQLKGS-C′ (280–296 amino acids).

### Culture and purification of dengue virus

Dengue virus serotype 2 (isolated from a human in 2005) was obtained from the Korean Bank for Pathogenic Viruses (KBPV-VR-29). The virus was infected into Vero cells (Monkey kidney cells) according to the instructions. Briefly, infection was allowed for 3 h and the cultured viral supernatant was collected at approximately 10 days post-infection, when a cytopathic effect was detected. The virus was inactivated with 0.3% formalin for 1 day at room temperature and pelleted using sucrose density gradient ultracentrifugation. Briefly, the inactivated virus suspension was laid on a 30–60% (w/v) isopycnic sucrose density gradient in a tube which fits an SW32TI ultracentrifuge rotor (Beckman Coulter Inc., Pasadena, CA, USA) and cold-centrifuged at 112,600 × g for 4 h. The titer was determined using a hemagglutination inhibition assay^38^ and at minimum, 2^8^ HAU of the virus was used for the subsequent experiment.

### Production and purification of monoclonal antibodies (mAbs)

Eight-week-old female BALB/c mice (DBL Inc., Eumseong, Chungbuk, Korea) were immunized by injecting 100 μg of peptide antigen conjugated with bovine serum albumin (BSA) at the C-terminus and the same volume of complete Freund’s adjuvant (Sigma-Aldrich Corp., St. Louis, MO, USA). After 2 weeks, a second injection that was prepared similarly, but mixed with incomplete Freund’s adjuvant (Sigma-Aldrich Corp.), was administered. A third injection was administered after another 2 weeks and the titer of anti-DENV EDI antibody in the serum was tested by ELISA (enzyme-linked immunosorbent assay) to determine whether an additional injection was required. Hybridoma cell fusion was performed as described previously^39^. Spleen cells (1 × 10^8^) were obtained and purified from immunized mice and fused with SP2/0 mouse myeloma cells (1 × 10^7^) (ATCC #CRL1581). Hybridomas producing specific mAbs were screened by an indirect ELISA using both peptide antigen and animal cell-cultured virus as coating antigens. Positive hybridomas were finally cloned by limiting dilution. Six- to eight-week-old female BALB/c mice (DBL Inc.) were injected with 0.5 ml of incomplete Freund’s adjuvant (Sigma-Aldrich Corp.). After 1 week, 0.5 ml of hybridomas (1.5 × 10^6^) was injected. Ascitic fluid of mice was isolated at 1–2 weeks and mixed with 10% (v/v) ammonium sulfate for 30 min at 4 °C. After centrifugation at 15,000 rpm for 30 min, the supernatant was separated, and the same process was repeated with 50% (v/v) ammonium sulfate. The pellet prepared by centrifugation was re-suspended with 20 mM phosphate buffer (pH 7.0), dialyzed using the same buffer for 18 hr (changing the buffer several times), and injected into a pre-equilibrated protein G-coupled Sepharose Column (GE Healthcare Life Science, Waukesha, WI, USA). mAbs were eluted with 10 mM glycine solution (pH 2.8) and directly neutralized with 1/10 volume of 1 M Tris buffer (pH 9.0). mAbs were further dialyzed after they were concentrated and stored at 4 °C until use.

### Determination of the isotype and affinity (*K*_d_) of mAbs

Isotypes of mAbs were determined using goat anti-mouse immunoglobulins (Sigma-Aldrich Corp.) as previously described^40^. Briefly, each isotype of the goat anti-mouse antibody was coated on the well. mAbs were added and incubated for 30–60 min. After washing, anti-mouse IgG-horseradish peroxidase conjugate was added and incubated for another 30–60 min. After a final washing, substrate solution (3,3′,5,5′-tetramethylbenzidine; TMB) was added to colorize the reaction. Absorbance was measured by a microplate reader (Benchmark Plus; Bio-Rad, Hercules, CA, USA) at 450 nm.

The affinity of mAbs was determined by an indirect competitive ELISA^41^. Various concentrations of peptide antigens or animal cell-cultured viruses were incubated with mAbs and then transferred to wells that were coated with peptide antigens or animal cell-cultured viruses. After incubation, wells were rinsed with phosphate-buffered saline containing 0.1% Tween-20. Wells were incubated with anti-mouse IgG-horseradish peroxidase conjugate and TMB solution was added after a final washing. The absorbance was measured by a microplate reader (Benchmark Plus, Bio-Rad) at 450 nm after terminating the reaction with H_2_SO_4_. The dissociation constant (*K*_d_) was calculated by generating a Klotz plot as described previously^41^.

### Western blot analysis

Ten micrograms of purified dengue virus and Hepatitis B virus (NAGASE & CO., LTD., Kyoto, Japan) were introduced to polyacrylamide gel and electrophoresed. A gel was transferred to a nitrocellulose membrane and the membrane was incubated with mAb specific for EDI (M5G6) for 2 h. After washing with Tris-buffered saline (pH 7.6), the membrane was incubated with goat anti-mouse IgG-HRP conjugate for 1 h. Immunoreactive band was visualized by an enhanced chemiluminescence detection system (Amersham Biosciences, Piscataway, NJ, USA).

### Preparation of RDT strips

Colloidal gold particles were prepared as previously described^42^. HAuCl_4_ (0.02%) was boiled in a beaker and 0.2% sodium citrate was added under constant stirring. When the solution turned wine-red in color, it was boiled for another 5 min and stirred for 10 min without boiling. The colloidal gold solution was stored in the dark at 4 °C before use. The mAb (1 mg) was conjugated with prepared colloidal gold particles (100 ml)^43^. The mAb-gold conjugate was precipitated by centrifugation and dissolved with phosphate-buffered saline containing 0.1% BSA to adjust the OD_450_ to 10. The conjugate was then treated on a glass fiber and dried to prepare the conjugator pad. The mAbs against human IgG and human IgM (Genbody Inc., Cheonan, Korea) were dispensed and immobilized at the appropriate positions (test lines 1 and 2, respectively) on a nitrocellulose membrane (0.5–4.0 mg/ml). Goat anti-mouse IgG (1 mg/ml) (Arista Biologicals Inc., Allentown, PA, USA) was dispensed and immobilized on the control line of the membrane. The buffer pad was prepared by treating cellulose paper (Grade 319; Ahlstrom Inc., Alpharetta, GA, USA) with 0.1 M Tris (pH 8.0). The absorbance pad consisted of untreated cotton paper. The dengue antigen pad was prepared by treating cellulose paper (Grade 6613; Ahlstrom Inc.) with untreated animal cell-cultured dengue virus serotype 2 (2^8^ HAU). The sample pad was prepared by treating an AP-22 cotton pad (Ahlstrom Inc.) with 0.05 M Tris (pH 8.0). All pads were partially overlapped to enable the migration of the sample and buffer solution along the strip.

### Clinical specimens

The Anti-Dengue Mixed Titer Performance Panel (SeraCare Life Science, Gaithersburg, MD, USA) was used in the comparative evaluation of RDTs. The panel consists of 21 undiluted, unpreserved plasma specimens displaying a range of reactivities for anti-dengue IgM and IgG antibodies, except for 1 member (panel no. 14). A total of 660 blood samples were provided by the Kuala Lumpur General Hospital, Malaysia, including 190 cases of anti-dengue IgM-positive, 320 cases of anti-dengue IgG-positive and 150 dengue-negative samples. All anti-dengue IgM- and IgG-positive samples were confirmed by ELISA (PanBio and SD BIOLINE capture ELISA). The use of the patient samples in this study was approved by the Institutional Review Board of the Kuala Lumpur General Hospital and the ethics committee. Written informed consent was also obtained from the patients who participated in this study. The experiments were carried out in accordance with the approved guidelines. Clinical specimens positive with Chikungunya virus (8), Tick-borne encephalitis virus (4), and yellow fever virus (3) were purchased from Bahiafarma Co. (Bahia, Brazil). Specimens positive with Japanese encephalitis virus (5) were purchased from AccoBiotech Inc. (Kuala Lumpur, Malaysia).

### Clinical evaluation of RDT

Ten microliters of specimen was loaded into the sample well of the device and 3 drops (~100 μl) of buffer solution were subsequently loaded into the buffer well of the device. Results were interpreted within 15 min. Tests were valid if a color appeared at the control line. If a red color appeared at the test line, the specimen contained anti-dengue IgG (test line 1), anti-dengue IgM (test line 2), or both (test line 1 and 2).

## Additional Information

**How to cite this article**: Lee, J. *et al.* Enhanced performance of an innovative dengue IgG/IgM rapid diagnostic test using an anti-dengue EDI monoclonal antibody and dengue virus antigen. *Sci. Rep.* **5**, 18077; doi: 10.1038/srep18077 (2015).

## Figures and Tables

**Table 1:** Properties of monoclonal antibodies developed in this study.

**Monoclonal antibody**	**Isotype**	**Affinity to envelope domain I (*****K***_d_*)	**Affinity to dengue virus (*****K***_d_*)
M5G6	IgG_1_	2.02 × 10^9^	1.82 × 10^9^
M1G10	IgG_1_	5.12 × 10^8^	1.51 × 10^8^
M1E4	IgG_2b_	5.23 × 10^8^	3.0 × 10^7^
M3F2	IgG_1_	1.12 × 10^9^	0.34 × 10^5^
M3G8	IgG_2a_	0.27 × 10^9^	NR**
M4D12	IgG_2a_	1.52 × 10^9^	NR**

^*^K_d_, Dissociation constant.

^**^NR, No response.

**Table 2:** Comparison of diagnostic results between SD and the kit developed in this study.

**Panel Member**	**Country of Origin**	**Anti-Dengue IgG**			**Anti-Dengue IgM**		
		**ELISA**^1^**(s/co**^3^)	**SD**^4^	**RDT in this study**	**ELISA**^2^**(s/co**^3^)	**SD**^4^	**RDT in this study**
PVD201-01	Colombia	>8.9	+	+	6.0	+	+
PVD201-02	Honduras	1.6	−	+	0.4 (−)	−	−
PVD201-03	Honduras	1.1	−	+	0.3 (−)	−	−
PVD201-04	Honduras	0.5 (−)	−	−	0.2 (−)	−	−
PVD201-05	Honduras	1.9	−	+	0.5 (−)	−	−
PVD201-06	Honduras	3.4	−	+	2.6	−	+
PVD201-07	Colombia	8.8	+	+	>7.7	+	+
PVD201-08	Honduras	1.2	−	+	1.0	−	−
PVD201-09	Honduras	2.1	−	+	1.2	−	+
PVD201-10	Ecuador	2.5	−	+	>7.7	+	+
PVD201−11	Honduras	1.0	−	−	0.4 (−)	−	−
PVD201-12	Honduras	0.7 (−)	−	−	0.4 (−)	−	−
PVD201-13	Honduras	2.1	−	+	0.6 (−)	−	−
PVD201-14*	USA	0.1 (−)	−	−	0.3 (−)	−	−
PVD201-15	Honduras	0.8 (−)	−	+	0.3 (−)	−	−
PVD201-16	Ecuador	2.4	+	+	6.8	+	+
PVD201-17	Honduras	>8.9	+	+	5.5	+	+
PVD201-18	Honduras	0.7 (−)	−	−	0.8 (−)	−	−
PVD201-19	Honduras	0.5 (−)	−	−	0.4 (−)	−	−
PVD201-20	Ecuador	3.0	+	+	>7.7	+	+
PVD201-21	Ecuador	>8.9	+	+	3.9	+	+
No. of positive		15	6	15	10	7	10
No. of negative		6	15	6	11	14	11
Sensitivity (%)			40	93.3		70	100
Specificity (%)			100	83.3		100	100

^*^Negative for anti-dengue; ^1^SD Dengue IgG Capture ELISA; ^2^SD Dengue IgM Capture ELISA; ^3^s/co (signal to cutoff ratio) ≥1.0 is considered reactive; ^4^SD BIOLINE Dengue IgG/IgM rapid kit.

**Table 3:** Clinical evaluation of the novel dengue IgG/IgM rapid test developed in this study.

		**ELISA**			**Total**
		**Anti-dengue IgG-positive**	**Anti-dengue IgM-positive**	**Dengue-negative**	
RDT	Anti-dengue IgG-positive	320	−	1*	321
	Anti-dengue IgM-positive	−	189		190
	Dengue-negative	0	1	149	150
Total		320	190	150	470

^*^both anti-dengue IgG- and anti-dengue IgM-positive.

**Table 4:** Field evaluation of the novel dengue IgG/IgM rapid test developed in this study.

			**Novel**	SD**	PanBio***
ELISA*	Anti-dengue IgM-positive	113	109 (36)#	62 (23)#	57 (27)#
	Anti-dengue IgG-positive	150	145	123	113
	Dengue-negative	57	57	57	57
Total		320	311	242	228
Sensitivity (%)	Anti-dengue IgM-positive		96.5 (31.9)	54.9 (20.4)	50.4 (23.9)
	Anti-dengue IgG-positive		96.7	82.0	75.3
Specificity (%)			100	100	100

^*^Positivity was double-checked by 2 independent ELISA tests (PanBio and SD BIOLINE).

^**^SD BIOLINE Dengue IgG/IgM (Alere Inc.).

^***^PanBio dengue Duo cassette (Alere Inc.).

^#^Secondary infection of dengue virus (anti-dengue IgG-positive).
